# What practicing pathologists and oncologists should know about the new computational pathology‐based companion diagnostic tools

**DOI:** 10.1002/path.70045

**Published:** 2026-02-25

**Authors:** Diana Montezuma, Sara P. Oliveira, Inti Zlobec, Nadieh Khalili, Jordi Temprana‐Salvador, Sabine Leh, David Ameisen, Mircea‐Sebastian Șerbănescu, Arsela Prelaj, Jakob Nikolas Kather, Norman Zerbe, Vincenzo L'Imperio

**Affiliations:** ^1^ European Society of Digital and Integrative Pathology (ESDIP) Lisbon Portugal; ^2^ Research & Development Unit, IMP Diagnostics Porto Portugal; ^3^ Cancer Biology and Epigenetics Group, Research Center of IPO Porto (CI‐IPOP) Portuguese Oncology Institute of Porto, Comprehensive Cancer Center Raquel Seruca (Porto.CCC) & CI‐IPOP@RISE (Health Research Network) Porto Portugal; ^4^ Computational Pathology Group, The Netherlands Cancer Institute Amsterdam The Netherlands; ^5^ Institute of Tissue Medicine and Pathology (ITMP), University of Bern Bern Switzerland; ^6^ Department of Digital Medicine University of Bern Bern Switzerland; ^7^ Computational Pathology Group, Department of Pathology Radboud University Medical Center Nijmegen The Netherlands; ^8^ Pathology Department, Vall Hebron University Hospital Barcelona Spain; ^9^ Department of Pathology Haukeland University Hospital Bergen Norway; ^10^ Department of Clinical Medicine University of Bergen Bergen Norway; ^11^ ImginIT Paris France; ^12^ Department of Medical Informatics and Biostatistics University of Medicine and Pharmacy of Craiova Craiova Romania; ^13^ European Interdisciplinary Society for AI in Cancer Research (ESAC) Milan Italy; ^14^ Thoracic Oncology Unit, Department of Medical Oncology, Fondazione IRCCS Istituto Nazionale dei Tumori Milan Italy; ^15^ Department of Electronics, Information, and Bioengineering Polytechnic University of Milan Milan Italy; ^16^ Else Kröner Fresenius Center for Digital Health, Faculty of Medicine TUD Dresden University of Technology Dresden Germany; ^17^ Department of Medicine I, Faculty of Medicine TUD Dresden University of Technology Dresden Germany; ^18^ Medical Oncology, National Center for Tumor Diseases (NCT) University Hospital Heidelberg Heidelberg Germany; ^19^ Pathology & Data Analytics, Leeds Institute of Medical Research at St James's University of Leeds Leeds UK; ^20^ Institute of Pathology RWTH Aachen University Hospital Aachen Germany; ^21^ Charité ‐ Universitätsmedizin Berlin, corporate member of Freie Universität Berlin and Humboldt Universität zu Berlin Institute of Medical Informatics Berlin Germany; ^22^ School of Medicine and Surgery University of Milano‐Bicocca Milan Italy; ^23^ Department of Pathology Fondazione IRCCS San Gerardo dei Tintori Monza Italy

**Keywords:** computational pathology, precision medicine, predictive oncology, digital pathology, TROP2, QCS, NMR

## Abstract

The integration of artificial intelligence into pathology is transforming the assessment of histological and immunohistochemical (IHC) slides, offering opportunities to reduce variability and streamline diagnostics. In practical terms, most available tools and research models emulate the diagnostic capabilities of pathologists by detecting, grading, and classifying tumours and other diseases. More recent applications have moved beyond mimicry, aiming to predict established biomarkers, such as microsatellite instability or IHC‐based markers, and to tackle even more ambitious tasks, such as directly predicting patient prognosis from H&E whole slide images. Remarkably, novel computational tools are now being designed as companion diagnostic assays, linking the automated evaluation of specific IHC biomarkers to the prediction of response to specific drugs, potentially marking a new chapter in the evolution of digital and computational pathology. The TROPION‐PanTumor01 trial recently demonstrated the superiority of a supervised machine learning model (termed the quantitative continuous score [QCS] by the vendor) in assessing TROP2 IHC compared with human scoring, promising better stratification of patients with non‐small cell lung cancer for treatment with datopotamab deruxtecan. The same approach has shown promise in refining HER2 (human epidermal growth factor receptor 2) and PD‐L1 (programmed death‐ligand 1) evaluations, revealing patient subgroups that may benefit from targeted therapies. Moreover, other similar approaches are progressively reaching the market, posing significant opportunities and challenges for clinicians involved in the care of patients with cancer. This Perspective is promoted by the European Society of Digital and Integrative Pathology (ESDIP, founded in 2016, and having long‐standing experience in computational pathology, esdipath.org) and the European Interdisciplinary Society of Artificial Intelligence for Cancer Research (ESAC, a recently established initiative, founded in 2024, esac-network.eu), both bringing together clinicians, engineers and other professionals dedicated to the development and clinical translation of computational approaches aimed at improving patient care. It aims to provide an informed overview of novel computational pathology companion diagnostic tools, with a particular focus on the background that practicing pathologists and oncologists need to have with these tools, when transitioning from research to clinical practice, irrespective of their prior familiarity with computational approaches. © 2026 The Author(s). *The Journal of Pathology* published by John Wiley & Sons Ltd on behalf of The Pathological Society of Great Britain and Ireland.

## Introduction

Digital pathology is progressively being adopted by laboratories worldwide, with a steadily increasing number of centres transitioning to digital workflows [1], as also documented by the recently available real‐time updated *Map of digital pathology labs*, tracking the digital transition of pathology institutions (https://www.esdipath.org/map-of-digital-pathology-labs/). The progressive digitisation of pathology laboratories is paving the way for the application of artificial intelligence (AI) tools to support routine tissue slide assessment. Beginning with workflow optimisation, such as prioritising urgent cases or cancer‐positive whole slide images (WSIs) [2], this technology holds the promise of reducing interobserver variability in several areas of pathology, such as tumour cell counting for molecular analysis [3, 4] and immunohistochemistry (IHC) interpretation [5]. It also opens the door to predicting the molecular landscape of tumours [6, 7] directly from digital images and identifying morphological prognostic markers that are otherwise invisible to the human eye [8, 9]. All of these applications require a fully digitised workflow [10] and still largely depend on traditional processing methods to generate physical H&E or IHC slides. Many also rely on established classification schemes and scoring systems, whose prognostic and predictive value have historically been validated through pathologists' assessments (e.g., HER2 [human epidermal growth factor receptor 2] scoring in breast cancer [11]). The progressive transition from ‘classic’ machine learning approaches to supervised and weakly supervised deep learning methods represents a further opportunity to extract quantitative information from digital histopathology images [12]. This was recently exemplified by the presentation of preliminary results from the TROPION‐Lung01 trial at the 2024 World Conference on Lung Cancer in San Diego, which represents a potential shift in the approach to biomarker discovery and precision oncology [13]. The trial introduced a new supervised deep learning method (termed quantitative continuous scoring [QCS] from AstraZeneca) as a predictive biomarker, which can potentially guide therapy with datopotamab deruxtecan (Dato‐DXd) in non‐small cell lung cancer (NSCLC) [13] by automatically measuring TROP2 (tumour‐associated calcium signal transducer 2) expression in tumour cells. This advancement in predictive pathology and oncology introduces valuable opportunities, while also underscoring the need for continued attention to validation, interpretability, and ethical considerations for clinicians who are expected to implement these tools in clinical practice. These issues are especially pressing given the emergence of IHC‐based predictive biomarkers (e.g., claudin 18.2 and FGFR2b in gastric and gastroesophageal adenocarcinoma [14] or folate receptor alpha in ovarian cancer [15, 16]), which may also benefit from similar computational interpretation approaches. In this context, the present Perspective is a joint initiative by the European Society of Digital and Integrative Pathology (ESDIP; https://www.esdipath.org) and the European Interdisciplinary Society of Artificial Intelligence for Cancer Research (ESAC; https://esac-network.eu/) aimed at summarising the current landscape of predictive computational biomarkers to promote awareness and education among clinicians, and providing a multidisciplinary perspective on the introduction of these tools.

## Revising the pathology biomarker world with computational approaches

The ability of AI technologies to predict various prognostic and theranostic markers (e.g., microsatellite instability [17] in colorectal cancer or epidermal growth factor receptor mutation in lung cancer [18]) directly from H&E‐stained WSIs is recognised, and their role within the diagnostic pathway of patients with cancer is progressively being defined. In particular, the ability to predict underlying genetic mutations, mostly imperceptible to the human eye on H&E slides, marks the onset of an AI‐driven precision pathology era, especially in light of the rise of foundation models [19], which integrate images and genomic data to further stratify patient outcomes and response to therapy. On a more practical and immediate level, some prognostic risk stratification systems are receiving CE‐IVD(R) (Conformité Européene In‐Vitro Diagnostic Regulation) approval, positioning them as alternatives to established molecular tests, particularly in areas such as breast pathology (e.g., Stratipath's or Owkin's predictive tools [20, 21]) and prostate cancer, where these are being progressively incorporated into patient management guidelines (namely ArteraAI Prostate Test [22]). Additionally, AI‐based assistants are becoming increasingly useful in helping pathologists to evaluate biomarkers that traditionally suffer from low interobserver reproducibility, but carry significant prognostic or predictive value, such as tumour‐infiltrating lymphocytes (TILs) in various cancers, including triple‐negative breast cancer [23]. Moreover, AI applied to pathology WSIs is uncovering previously overlooked morphological features, such as the tumour‐adipose feature (TAF) in colorectal cancer [9]. TAF is now emerging as an independent prognostic factor across gastrointestinal tumours and is an example of an AI‐facilitated biomarker discovery that can be easily translated into patterns/features recognisable by pathologists [8]. While these AI‐informed biomarkers are mainly based on H&E, there are already examples of commercially available algorithms that can assist in the interpretation of various IHC stainings (e.g., PD‐L1 (programmed death‐ligand 1), either as tumour proportion score (TPS) [24] or combined positive score [25], in different settings and HER2 in breast [26]), alleviating the burden on pathologists in time‐consuming and poorly reproducible tasks, on which oncologists rely for the prescription of therapies. Although these advances promise to significantly enhance pathology and oncology workflows, it is important to note that they are currently intended as adjuncts to the pathologist's evaluation. Their primary role is to streamline diagnostic processes, reduce variability, and provide information on patient‐related outcomes, serving as a support tool for pathologists rather than directly predicting responses to specific therapeutic agents. In contrast, the new computational pathology companion diagnostic tools represent a novel approach, integrating fully automated algorithms specifically designed to target specific biomarkers and predict responses to their corresponding therapeutic agents. These new tools surpass human scoring capabilities, as they can extract and quantify subtle spatial and morphological data that human observers cannot, due to perceptual limitations.

## Computational pathology tools as companion diagnostic assays

**Figure 1:**
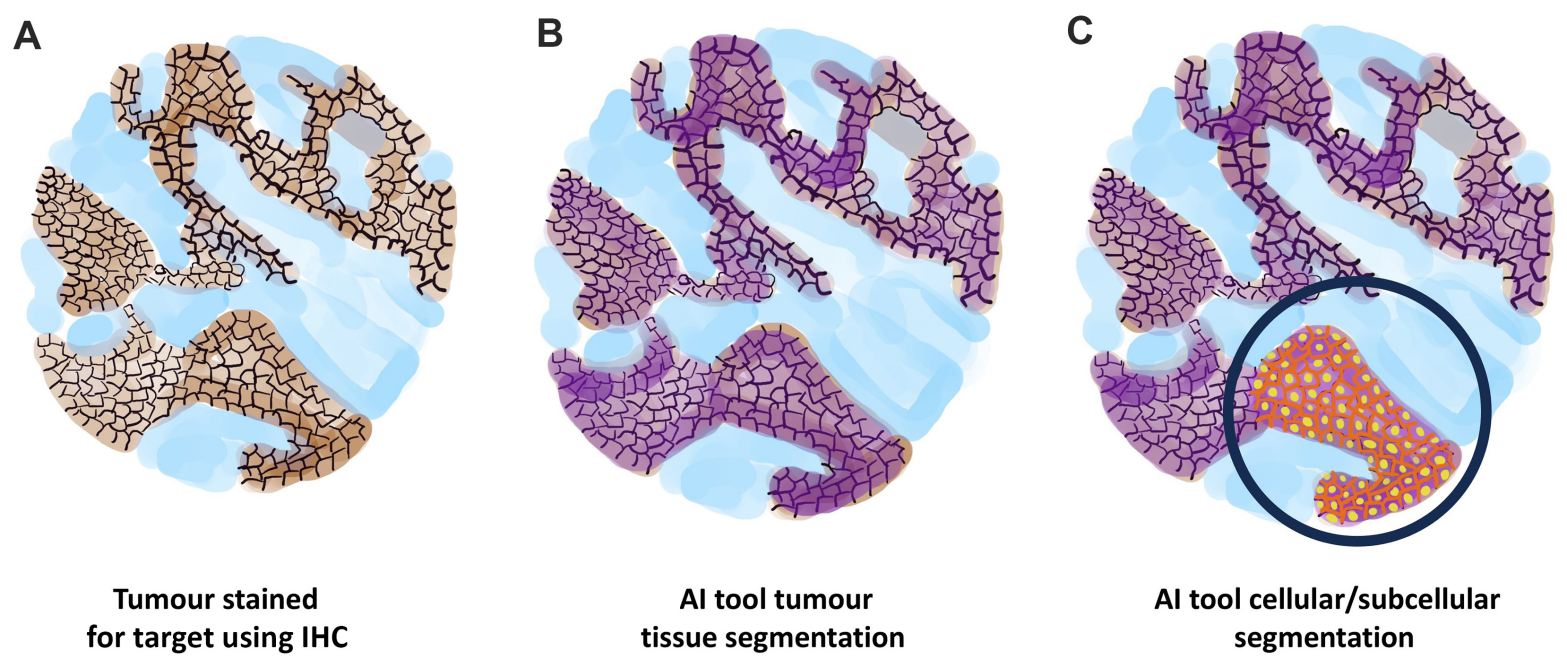
Schematic representation of the segmentation steps in a novel computational pathology tool. From left to right: (A) the tissue slide containing the tumour is immunostained with the specific marker of interest and scanned; (B) the whole slide image (WSI) is analysed with the deep learning method for tumour cell detection; and (C) subsequent cellular and subcellular compartment identification. Within the tumour cells, the artificial intelligence (AI) algorithm quantifies specific parameters (e.g., optical density), which can then be used to generate scores correlating with response or resistance to therapies. IHC, immunohistochemistry.

The TROPION‐PanTumor01 trial (https://www.clinicaltrials.gov/study/NCT03401385) recently evaluated the efficacy of Dato‐DXd, an antibody–drug conjugate, in NSCLC. Notably, the study demonstrated no clear correlation between TROP2 expression levels (assessed by IHC and interpreted by pathologists using the H‐score [27]) and the clinical response to Dato‐DXd treatment [27]. This evidence has prompted the search for alternative strategies to assess biomarker expression based on mechanisms of action of the drug, leading to the application of a supervised deep learning method (termed QCS) for the analysis of IHC‐stained slides. This algorithm was trained based on expert pathologist annotations to identify tumour versus other tissues and subcellular compartments (namely the cell membrane and cytoplasm) at single‐cell resolution across the entire WSI (Figure 1) [13]. Following this segmentation, the algorithm quantifies the intensity of the signal (by optical density, OD) produced by the brown chromogen used in IHC (DAB). This step is performed at both the cellular and subcellular levels, generating continuous, quantitative measurements of biomarker expression for each cell (further details on this specific type of algorithm can be found in the vendor instructions [28]). These measurements are then summarised across regions of interest, yielding metrics, such as percentage of cells above a defined cutoff, or used directly as continuous variables, offering a more nuanced and reproducible alternative to traditional categorical scoring [29].

The application of such a method in the setting of the TROPION‐PanTumor01 trial allowed the identification of a subset of patients with higher objective response rate (32.7% versus 10.3%) and longer median progression‐free survival (PFS), characterised by ≥75% tumour cells with ≤ 0.56 normalised membrane ratio (NMR, defined as membrane OD/[membrane+cytoplasm OD]) [13]. It is important to note that it is not possible for human observers to objectively quantify these parameters due to the subtle differences in membrane staining intensity across thousands of cells. Similar supervised deep learning methods have also been applied to HER2 interpretation in breast cancer [29, 30], identifying a higher proportion of HER2‐positive cases compared with manual scoring (76.4% versus 56.9%) [29], uncovering a subset of HER2‐ultralow cases missed by pathologists (23.6% of cases) [31]. Analogous results were obtained for PFS and overall survival (OS) when analysing HER2 in patients with colorectal [32] and gastric [33] cancer. Finally, these methods have already shown promising results in the evaluation of PD‐L1 IHC in lung cancer (TPS), outperforming pathologists in the stratification of OS based on immunotherapy [34]. As new IHC‐based predictive biomarkers continue to emerge across cancer types (such as Claudin 18.2 and FGFR2b in gastric and gastroesophageal adenocarcinomas [14] or folate receptor alpha in ovarian cancer [15, 16]), these novel computational approaches are positioned to deliver significant benefits, but also challenges. Importantly, their application may further transform the oncological biomarker landscape, enabling more precise, reproducible and scalable patient stratification in an increasingly complex therapeutic environment.

## What's next? Dealing with the challenges in the implementation of these AI‐based tools

The introduction of such computational pathology pipelines poses several challenges (Table 1). From a practical and technical standpoint, the rigorous standardisation of the pre‐analytical phase is essential to prevent artifacts (such as tissue folds, out‐of‐focus regions, or air bubbles) that could compromise algorithmic interpretation [35]. In this context, a systematic revision of workflow processes within pathology laboratories is critical to ensure reliability and reproducibility of such predictive biomarker assessments [10]. Additionally, because these methods rely on IHC‐stained WSIs, it is crucial to minimise variability introduced by different antibody clones, staining platforms, and detection systems. This ensures equitable access to accurate testing and therapeutic decisions, regardless of the laboratory or institution performing the analysis. To address these concerns, the first IHC‐based computational predictive tool (TROP2 QCS‐NMR) was recently granted breakthrough device designation by the US Food and Drug Administration (FDA) as a companion diagnostic [36]. This designation reflects the comprehensive integration of the antibody clone, staining platform, WSI scanner, and algorithmic pipeline, all standardised and provided by a single vendor, ensuring consistency and clinical‐grade performance across sites [36]. Although this ensures standardisation of the results [37] and equal access to treatments, it also requires significant investment and potential modifications within laboratories to comply with these standards, or the outsourcing of testing. As an alternative, ‘opening’ these locked testing pipelines to allow more open and flexible combinations (e.g., stainer/scanners/algorithms from different vendors) after adequate harmonisation of the comparative results would represent an alternative to allow the implementation of such tests in as many laboratories as possible. Another challenge is the limited interpretability and explainability of AI decisions in this context. The calculations of these supervised deep learning methods rely on colorimetric features of cellular and subcellular compartments. While the algorithm's output can still be evaluated by the human eye, the final result of the computation and its correspondence to the visual output cannot be verified by pathologists, potentially rendering the computation a black box. This limitation may hinder the ability of pathologists and clinicians to independently verify the reliability of AI‐derived results [38]. In pathology, we are already used to not evaluating results from molecular pathology tests but rather relying on robust assay‐wide validation, trusting their performance within their intended clinical context. Similarly, computational biomarkers can be evaluated based on the strength of their validation studies, rather than on the interpretability of each output. Moreover, the integration of these tools (particularly in a companion diagnostic setting) could be significantly enhanced by the establishment of specific reimbursement codes. In this regard, it is crucial to involve all relevant stakeholders (industry, pathologists, clinicians, and policy regulators) to enable the successful adoption of these tools in clinical practice. Lastly, ethical and regulatory considerations must be addressed before routine adoption. These include clearly defining liability in cases of inappropriate treatment indications due to pre‐analytical or post‐analytical errors, and mitigating the risk of biased outcomes from computational biomarkers, bearing in mind the intrinsic limitations of other human‐interpreted biomarkers on which precision oncology has historically been based on. Recent literature indicates that when AI systems move beyond a purely supportive role and begin to substitute elements of human decision‐making, the attribution of responsibility becomes considerably more complex, potentially extending beyond healthcare professionals to include developers and manufacturers of AI technologies [39]. However, this remains an evolving area, and further legal, ethical, and regulatory research is needed to clearly define liability frameworks in increasingly autonomous AI‐driven diagnostic settings, especially in light of the new directives introduced with the European AI Act [40].

**Table 1:** Challenges to address with next‐generation computational pathology tools.

1. Pre‐analytical variability (e.g., tissue folds, air bubbles, out‐of‐focus regions).
2. Lack of workflow integration and standard operating procedures in pathology labs.
3. Variability in immunohistochemistry (IHC) staining (antibody clones, platforms, detection systems).
4. High financial and infrastructure investment requirements for artificial intelligence (AI) adoption.
5. Limited explainability and interpretability of AI‐based decisions.
6. Reduced pathologist engagement due to the black box nature of AI.
7. Lack of performance and interpretation guidelines.
8. Absence of specific reimbursement codes for computational diagnostics.
9. Gap between the creation and introduction of these innovative tools.
10. Ethical concerns: liability, algorithmic bias, and misuse of prediction.

## Conclusions

While computationally derived tissue‐based biomarkers offer great promise for precision oncology, they challenge traditional clinical workflows. A multidisciplinary approach involving pathologists, oncologists, computer scientists, and regulators is essential to ensure safe and effective integration. Practicing pathologists and oncologists, regardless of prior experience with computational tools, should be aware of these next‐generation technologies. Although these tools still require further validation, they may support clinical decision‐making in the near future.

## Author contributions statement

DM and SPO contributed equally to this work. DM, VL and SPO conceptualised the manuscript and coordinated contributions. IZ, NK, JTS, SL, M‐SS, AP, DA, JNK and NZ critically reviewed the content and provided clinical and technical expertise. All authors contributed to writing, read, and approved the final manuscript.

## Data Availability

Data sharing not applicable to this article as no datasets were generated or analysed during the current study.

## References

[1] Pinto DG , Bychkov A , Tsuyama N , *et al*. Real‐world implementation of digital pathology: results from an intercontinental survey. Lab. Investig. 2023; 103 **:** 100261. 37839634 10.1016/j.labinv.2023.100261

[2] Rienda I , Vale J , Pinto J , *et al*. Using artificial intelligence to prioritize pathology samples: report of a test drive. Virchows Arch. 2025; 487 **:** 203–208. 39627613 10.1007/s00428-024-03988-1

[3] L'Imperio V , Cazzaniga G , Mannino M , *et al*. Digital counting of tissue cells for molecular analysis: the QuANTUM pipeline. Virchows Arch. 2025; 486 **:** 277–286. 38532196 10.1007/s00428-024-03794-9 PMC11876257

[4] Frei AL , Oberson R , Baumann E , *et al*. Pathologist computer‐aided diagnostic scoring of tumor cell fraction: a Swiss National Study. Mod. Pathol. 2023; 36 **:** 100335. 37742926 10.1016/j.modpat.2023.100335

[5] Brattoli B , Mostafavi M , Lee T , *et al*. A universal immunohistochemistry analyzer for generalizing AI‐driven assessment of immunohistochemistry across immunostains and cancer types. NPJ Precis Oncol 2024; 8 **:** 277. 39627299 10.1038/s41698-024-00770-z PMC11615360

[6] Echle A , Ghaffari Laleh N , Quirke P , *et al*. Artificial intelligence for detection of microsatellite instability in colorectal cancer‐a multicentric analysis of a pre‐screening tool for clinical application. ESMO Open 2022; 7 **:** 100400. 35247870 10.1016/j.esmoop.2022.100400 PMC9058894

[7] Fremond S , Andani S , Barkey Wolf J , *et al*. InterpreTable deep learning model to predict the molecular classification of endometrial cancer from haematoxylin and eosin‐stained whole‐slide images: a combined analysis of the PORTEC randomised trials and clinical cohorts. Lancet Digit Health 2023; 5 **:** e71–e82. 36496303 10.1016/S2589-7500(22)00210-2

[8] Reitsam NG , Grosser B , Steiner DF , *et al*. Converging deep learning and human‐observed tumor‐adipocyte interaction as a biomarker in colorectal cancer. Commun. Med. (Lond.) 2024; 4 **:** 163. 39147895 10.1038/s43856-024-00589-6 PMC11327259

[9] L'Imperio V , Wulczyn E , Plass M , *et al*. Pathologist validation of a machine learning‐derived feature for colon cancer risk stratification. JAMA Netw. Open 2023; 6 **:** e2254891. 36917112 10.1001/jamanetworkopen.2022.54891 PMC10015309

[10] Fraggetta F , L'Imperio V , Ameisen D , *et al*. Best practice recommendations for the implementation of a digital pathology workflow in the anatomic pathology laboratory by the European Society of Digital and Integrative Pathology (ESDIP). Diagnostics (Basel) 2021; 11 **:** 2167. 34829514 10.3390/diagnostics11112167 PMC8623219

[11] Wolff AC , Somerfield MR , Dowsett M , *et al*. Human epidermal growth factor receptor 2 testing in breast cancer: ASCO‐College of American pathologists guideline update. J. Clin. Oncol. 2023; 41 **:** 3867–3872. 37284804 10.1200/JCO.22.02864

[12] Shmatko A , Ghaffari Laleh N , Gerstung M , *et al*. Artificial intelligence in histopathology: enhancing cancer research and clinical oncology. Nat. Can. 2022; 3 **:** 1026–1038. 10.1038/s43018-022-00436-4 36138135

[13] Garassino MC , Sands J , Paz‐Ares L , *et al*. Normalized membrane ratio of TROP2 by quantitative continuous scoring is predictive of clinical outcomes in TROPION‐lung 01. J. Thorac. Oncol. 2024; 19 **:** S2–S3.

[14] Caputo A , Angerilli V , Gambella A , *et al*. Immunohistochemical biomarker scoring in gastroesophageal cancers: can computers help us? Pathol. Res. Pract. 2025; 272 **:** 156068. 40472683 10.1016/j.prp.2025.156068

[15] Matulonis UA , Lorusso D , Oaknin A , *et al*. Efficacy and safety of Mirvetuximab Soravtansine in patients with platinum‐resistant ovarian cancer with high folate receptor alpha expression: results from the SORAYA study. J. Clin. Oncol. 2023; 41 **:** 2436–2445. 36716407 10.1200/JCO.22.01900 PMC10150846

[16] Moore KN , Oza AM , Colombo N , *et al*. Phase III, randomized trial of mirvetuximab soravtansine versus chemotherapy in patients with platinum‐resistant ovarian cancer: primary analysis of FORWARD I. Ann. Oncol. 2021; 32 **:** 757–765. 33667670 10.1016/j.annonc.2021.02.017

[17] Kather JN , Pearson AT , Halama N , *et al*. Deep learning can predict microsatellite instability directly from histology in gastrointestinal cancer. Nat. Med. 2019; 25 **:** 1054–1056. 31160815 10.1038/s41591-019-0462-y PMC7423299

[18] Campanella G , Kumar N , Nanda S , *et al*. Real‐world deployment of a fine‐tuned pathology foundation model for lung cancer biomarker detection. Nat. Med. 2025; 31 **:** 3002–3010. 40634781 10.1038/s41591-025-03780-x PMC12443599

[19] Vaidya A , Zhang A , Jaume G , *et al*. Molecular‐driven foundation model for oncologic pathology. *arXiv* 2025; 2501.16652. [Not peer reviewed].

[20] Sharma A , Lövgren SK , Eriksson KL , *et al*. Validation of an AI‐based solution for breast cancer risk stratification using routine digital histopathology images. Breast Cancer Res. 2024; 26 **:** 123. 39143539 10.1186/s13058-024-01879-6 PMC11323658

[21] Garberis I , Gaury V , Saillard C , *et al*. Deep learning assessment of metastatic relapse risk from digitized breast cancer histological slides. Nat. Commun. 2025; 16 **:** 5876. 40593633 10.1038/s41467-025-60824-z PMC12216745

[22] Armstrong AJ , Liu VYT , Selvaraju RR , *et al*. Development and validation of an artificial intelligence digital pathology biomarker to predict benefit of long‐term hormonal therapy and radiotherapy in men with high‐risk prostate cancer across multiple phase III trials. J. Clin. Oncol. 2025; 43 **:** 3494–3504. 40239134 10.1200/JCO.24.00365 PMC12228211

[23] Vidal JM , Tsiknakis N , Staaf J , *et al*. The analytical and clinical validity of AI algorithms to score TILs in TNBC: can we use different machine learning models interchangeably? EClinicalMedicine 2024; 78 **:** 102928. 39634035 10.1016/j.eclinm.2024.102928 PMC11615110

[24] Plass M , Olteanu G‐E , Dacic S , *et al*. Comparative performance of PD‐L1 scoring by pathologists and AI algorithms. Histopathology 2025; 87 **:** 90–100. 39961605 10.1111/his.15432 PMC12129605

[25] Bossard C , Magois C , Roussel H , *et al*. Clinical evaluation of an automated pan‐organ combined PD‐L1 scoring using artificial intelligence on immunostained whole‐slide images. ESMO Real World Data and Digital Oncology 2025; 10 **:** 100181.

[26] Krishnamurthy S , Schnitt SJ , Vincent‐Salomon A , *et al*. Fully automated artificial intelligence solution for human epidermal growth factor receptor 2 immunohistochemistry scoring in breast cancer: a multireader study. JCO Precis. Oncol. 2024; 8 **:** e2400353. 39393036 10.1200/PO.24.00353 PMC11485213

[27] Shimizu T , Sands J , Yoh K , *et al*. First‐in‐human, phase I dose‐escalation and dose‐expansion study of trophoblast cell‐surface antigen 2‐directed antibody‐drug conjugate Datopotamab Deruxtecan in non‐small‐cell lung cancer: TROPION‐PanTumor01. J. Clin. Oncol. 2023; 41 **:** 4678–4687. 37327461 10.1200/JCO.23.00059 PMC10564307

[28] Reis‐Filho JS , Scaltriti M , Kapil A , *et al*. Shifting the paradigm in personalized cancer care through next‐generation therapeutics and computational pathology. Mol. Oncol. 2024; 18 **:** 2607–2611. 39214683 10.1002/1878-0261.13724 PMC11547233

[29] Kapil A , Spitzmüller A , Brieu N , *et al*. HER2 quantitative continuous scoring for accurate patient selection in HER2 negative trastuzumab deruxtecan treated breast cancer. Sci. Rep. 2024; 14 **:** 12129. 38802399 10.1038/s41598-024-61957-9 PMC11130140

[30] Robbins CJ , Bates KM , Rimm DL . HER2 testing: evolution and update for a companion diagnostic assay. Nat. Rev. Clin. Oncol. 2025; 22 **:** 408–423. 40195456 10.1038/s41571-025-01016-y PMC12903097

[31] Lee T , Shin S , Brattoli B , *et al*. Identification of HER2 ultra‐low based on an artificial intelligence (AI)‐powered HER2 subcellular quantification from HER2 immunohistochemistry images. J Clin Oncol 2024; 42 **:** 1115.

[32] Nakamura Y , Okamoto W , Kato T , *et al*. Artificial intelligence (AI)‐powered HER2 quantification continuous score (QCS) and tumor microenvironment (TME) analysis in HER2‐amplified metastatic colorectal cancer (mCRC) treated with pertuzumab plus trastuzumab. JCO Glob Oncol 2023; 9 **:** 34.

[33] Kapil A , Failmezger H , Spitzmüller A , *et al*. Computational pathology–based HER2 quantification to identify novel biomarkers in gastric cancer (GC). J Clin Oncol 2023; 41 **:** 449.

[34] Schmidt G , Kapil A , Meinecke L , *et al*. Computational pathology delivers objective and quantitative PD‐L1 expression analysis for enrichment of responders to durvalumab in non‐small cell lung cancer (NSCLC). J. Immunother. Cancer 2021; 9 **:** A1–A1054.

[35] Weng Z , Seper A , Pryalukhin A , *et al*. GrandQC: a comprehensive solution to quality control problem in digital pathology. Nat. Commun. 2024; 15 **:** 10685. 39681557 10.1038/s41467-024-54769-y PMC11649692

[36] Roche granted FDA Breakthrough Device Designation for first AI‐driven companion diagnostic for non‐small cell lung cancer. [Accessed June 10, 2025]. Available from: https://www.roche.com/investors/updates/inv-update-2025-04-29.

[37] Lopez‐Rios F , Inge LJ , Blechet C , *et al*. Real‐world assessment of TROP2‐NMR by quantitative continuous scoring (QCS) in non‐small cell lung carcinoma (NSCLC). J. Thorac. Oncol. 2025; 20 **:** S29–S30.

[38] Acs B , Fend F , Guettier C , *et al*. Debating the pros and cons of computational pathology at the European Congress of Pathology (ECP) 2024. Virchows Arch. 2025. 10.1007/s00428-025-04084-8. 40131426

[39] Mobilio G , Orlandi E , Del Riccio M , *et al*. Should healthcare resist AI?: the transformative impact of LLMs in digital pathology. In *Proceedings of the* 2025 *19th International Symposium on Medical Information and Communication Technology (ISMICT)* . IEEE, 2025; 1–6.

[40] Deman F , Palmieri S , Broeckx G , *et al*. Practical consequences of the European union‐AI act for anatomic pathology laboratories a European Society of Pathology and European Society of Digital and Integrative Pathology commissioned expert opinion paper. Virchows Arch 2025. 10.1007/s00428-025-04291-3. PMC13053491 41102487

